# Doing Better by Getting Worse: Posthypnotic Amnesia Improves Random Number Generation

**DOI:** 10.1371/journal.pone.0029206

**Published:** 2011-12-15

**Authors:** Devin Blair Terhune, Peter Brugger

**Affiliations:** 1 Department of Experimental Psychology, University of Oxford, Oxford, United Kingdom; 2 Department of Neurology, University Hospital Zurich and Zurich Center of Integrative Human Physiology (ZIHP), University of Zurich, Zurich, Switzerland; Royal Holloway, University of London, United Kingdom

## Abstract

Although forgetting is often regarded as a deficit that we need to control to optimize cognitive functioning, it can have beneficial effects in a number of contexts. We examined whether disrupting memory for previous numerical responses would attenuate repetition avoidance (the tendency to avoid repeating the same number) during random number generation and thereby improve the randomness of responses. Low suggestible and low dissociative and high dissociative highly suggestible individuals completed a random number generation task in a control condition, following a posthypnotic amnesia suggestion to forget previous numerical responses, and in a second control condition following the cancellation of the suggestion. High dissociative highly suggestible participants displayed a selective increase in repetitions during posthypnotic amnesia, with equivalent repetition frequency to a random system, whereas the other two groups exhibited repetition avoidance across conditions. Our results demonstrate that temporarily disrupting memory for previous numerical responses improves random number generation.

## Introduction

Although forgetting is often regarded as a deficit that we need to control to optimize cognitive functioning, it can have beneficial effects in a number of contexts [1]. One such instance may be when memory for previous responses reduces spontaneity in subsequent responding. There is reason to believe that this is the case with biases in random number generation (RNG) [2], [3]. Despite the compelling intuition that generating strings of random numbers is relatively easy, human beings are notoriously poor at randomizing a set of alternatives [2]. In RNG tasks, individuals frequently avoid repeating the same number (*repetition avoidance*) and tend to arrange consecutive numbers in an ascending or descending order (*counting bias*) more often than a random system [4]. It is recognized that taxing memory by increasing memory load or prolonging the inter-response interval improves random number generation [2], [3]. However, these approaches are confounded by the fact that they also eliminate the rapidity of a fast pace, which will also reduce stereotyped responding. In this study, we tested the prediction that disrupting memory for previous numerical responses with a suggestion for posthypnotic amnesia would attenuate response bias during RNG.

Posthypnotic amnesia involves a suggestion to forget some type of information following hypnosis and can be strikingly effective at disrupting recognition and recall of both semantic and episodic information in highly suggestible (HS) individuals [5]–[7], who make up approximately 10–15% of the population [8]. The suggestion can also be subsequently cancelled, permitting a return to normal mnemonic functioning. Proneness to dissociative states such as depersonalization is associated with greater responsiveness to posthypnotic suggestions among HS individuals [9], [10]. We predicted that posthypnotic amnesia for one's previous responses would attenuate response biases in RNG in high dissociative (HDHS), but not low dissociative (LDHS), individuals.

## Materials and Methods

### Ethics statement

All participants provided informed written consent and all procedures were performed in accordance with the approval of the Swedish Federal Human Subjects Agency (Etikprövningsnämden).

### Participants

Eight low suggestible (LS) and twelve HS individuals, drawn from a sample of over 600 individuals [11], participated in this experiment. Hypnotic suggestibility was initially measured in group sessions with the *Waterloo-Stanford Group Scale of Hypnotic Susceptibility*, *Form C* (WSGC) [12] and corroborated in individual sessions with the *Revised Stanford Profile Scales of Hypnotic Susceptibility* (RSPSs) [13]. The eight LS and 12 HS participants met criteria for low and high hypnotic suggestibility, respectively (LS: WSGCS≤4, RSPS≤4; HS: WSGC≥8, RSPSs≥20) [11]. In a non-hypnotic context, participants completed the *Swedish Dissociative Experiences Scale* [14], which indexes an individual's propensity for experiencing episodes of dissociation. LDHS (*n* = 8, *M* = 11.87, *SD* = 4.81) and HDHS (*n* = 4, *M* = 28.30, *SD* = 4.24) were identified using a cut-off criterion of 20 for establishing high dissociation, which corresponded to the 75^th^ percentile in a mixed-sample of LS and HS individuals [11] (this is a widely used criterion for establishing high dissociation, see [15]); LS participants were uniformly low in dissociation (*M* = 8.26, *SD* = 5.84). LS (six female; *M*_Age_ = 23.38, *SD* = 3.38), LDHS (six female; *M*_Age_ = 26.13, *SD* = 2.17), and HDHS (three female; *M*_Age_ = 25.00, *SD* = 2.16), participants did not differ in sex distributions, χ^2^(2) = 0, or age, *F*(2, 17)<2.1.

### Stimuli and procedure

We measured RNG by having participants verbally respond to 50 ms 1 Hz auditory tones with 5000 ms interstimulus intervals with a random number from the range of 1 to 6 [2]. Participants completed 66 trials at baseline (control condition) and were then administered a hypnotic induction and the posthypnotic amnesia suggestion:

In a few moments I will dehypnotize you by counting backwards from 10 to 1. At 1, you will open your eyes and be wide-awake. Shortly afterwards, I'm going to ask you to complete the same number task that you did before. However, when you perform the task this time, you will find that whenever you hear one of the auditory tones you will immediately forget the last number that you stated and all of the numbers that came before it. Forgetting your previous responses will not affect your ability to state numbers when you hear the auditory tones. You will remain this way until I say “Okay, you can remember previous numbers now” [post-cancellation cue]. When I say those words you will again be able to remember what happened during hypnosis as well as the numbers you stated prior to each auditory tone.

The experimenter then administered a hypnotic de-induction and participants completed the task a second time (posthypnotic amnesia condition) and once more after the cancellation of the suggestion (post-cancellation control condition). Upon completion of the latter condition, participants provided self-reports regarding the magnitude of forgetting of previous responses in the RNG task during the posthypnotic amnesia condition relative to the post-cancellation control condition (1 = no forgetting to 4 = complete forgetting); this score was used as a measure of self-perceived magnitude of response to the posthypnotic amnesia suggestion.

RNG performance was evaluated by the analysis of first-order differences (FODs) computed from sequential responses. The analyses were focused on repetitions (FOD = 0) and descending and ascending counting (FOD = −1 or +1, respectively). In order to evaluate whether participants' responses deviated from random responding, we also contrasted participants' FODs with FODs computed from a single set of 1000 simulated vectors of 66 random numbers from the range 1 to 6 (*simulated data*).

## Results

Self-reports of the perceived magnitude of forgetting of previous responses during the completion of the RNG task in the posthypnotic amnesia condition were analyzed with a Kruskal-Wallis test because the data violated the assumption of homogeneity of variance. This analysis revealed a main effect of Group, *H*(2) = 15.63, *p*<.001, η_p_^2^ = .82. *Post hoc* Mann-Whitney tests indicated that LS participants reported no forgetting (*M* = 1, *SD* = 0), which was significantly less than the pronounced forgetting reported by LDHS (*M* = 3, *SD* = 0.76, range: 2 to 4), *U* = 0, *Z* = 3.63, *p*<.001, *d* = 3.98, and by HDHS (*M* = 3.5, *SD* = 0.58, range: 3 to 4), *U* = 0, *Z* = 3.25, *p* = .001, *d* = 8.62, who did not differ, *t*(10)<1.2.

**Figure 1:**
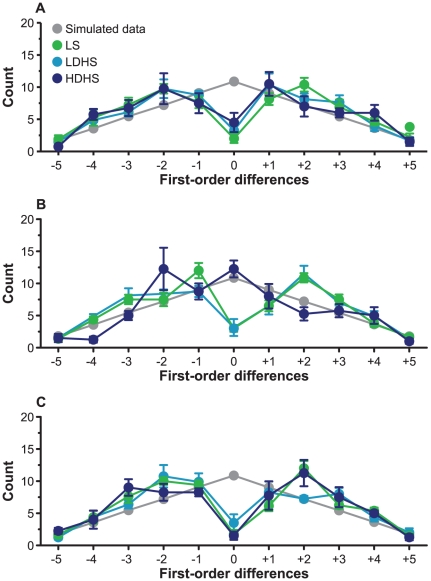
First-order difference counts during a serial RNG task. The data (*M* ± *SEM*) are presented in (A) control, (B) posthypnotic amnesia, and (C) post-cancellation conditions in the three participant groups and in simulated data. LS = low suggestible; LDHS = low dissociative highly suggestible; HDHS = high dissociative highly suggestible.

Repetition avoidance (reduced FOD 0 counts relative to the simulated data) can be seen in Figure 1. A 3 (Condition)×3 (Group) mixed-model ANOVA on FOD 0 counts (repetitions) revealed a main effect of Condition, *F*(2, 34) = 40.66, *p*<.001, η_p_^2^ = .71, and a suggestive main effect of Group, *F*(2, 17) = 3.09, *p* = .072, η_p_^2^ = .27, which were qualified by a Condition×Group interaction, *F*(4, 34) = 25.90, *p*<.001, η_p_^2^ = .75. Neither LS, *F*(2, 14)<0.5, nor LDHS, *F*(2, 14)<2.6, participants differed across conditions, whereas HDHS participants did, *F*(2, 6) = 36.02, *p*<.001, η_p_^2^ = .92. As predicted, HDHS participants produced more repetitions in the posthypnotic amnesia condition than in the two control conditions, planned contrasts: *F*s(1, 3)>34, *p*s≤.01, η_p_^2^s>.91, which did not differ, *t*(3)<2.8. Subsidiary analyses revealed that HDHS participants were also more repetitive than LS, *t*(10) = 4.35, *p* = .001, *d* = 2.91, and LDHS, *t*(10) = 7.88, *p*<.001, *d* = 5.29, participants in the posthypnotic amnesia condition, but in neither of the control conditions, *t*s(10)<1.8. LS and LDHS participants did not differ in any of the conditions, *t*s (14)<1.2. Relative to the simulated data, LS, *t*s(1006)>6.9, *p*s<.001, *d*s>2.4, and LDHS, *t*s(1006)>7.4, *p*s<.001, *d*s>2.6, participants exhibited fewer repetitions in all three conditions, demonstrating persistent repetition avoidance. In contrast, HDHS participants displayed repetition avoidance in the two control conditions, *t*s(1002)>4.2, *p*s<.001, *d*s>2.1, but not in the posthypnotic amnesia condition, *t*(1002)<1. These results point to a selective increase in repetitions in the posthypnotic amnesia condition that was only present in HDHS participants. Critically, HDHS participants' FOD 0 counts in this condition were indistinguishable from the output of a random system.

A mixed-model ANOVA on FOD −1 counts (descending counting bias) revealed a main effect of Condition, *F*(2, 34) = 3.92, *p* = .029, η_p_^2^ = .19, but no main effect of Group, *F*(2, 17)<1, and a Condition×Group interaction, *F*(4, 34) = 2.79, *p* = .042, η_p_^2^ = .25. Subsidiary analyses showed that LDHS participants differed across conditions, *F*(2, 14) = 7.23, *p* = .007, η_p_^2^ = .51, whereas neither LS, *F*(2, 14)<1, nor HDHS, *F*(2, 6)<1, did. *Post hoc* contrasts showed that LDHS participants displayed greater FOD −1 counts in the posthypnotic amnesia condition than in the control condition, *t*(7) = 3.76, *p* = .007, *d* = 1.57, but not in the post-cancellation condition, *t*(7)<2.25; the latter two conditions did not differ, *t*(7)<1.8. LDHS participants' FOD −1 counts were greater than the counts in the simulated data in the posthypnotic amnesia condition, *t*(1006) = 2.88, *p* = .004, *d* = 1.02, but in neither of the control conditions, *t*s(1006)<1.5; the counts of LS, *t*s(1006)<1, and HDHS, *t*s(1002)<1.2, participants didn't differ from the counts in the simulated data in any of the conditions. These results indicate that LDHS participants displayed an increase in the descending counting bias during the posthypnotic amnesia condition.

A mixed-model ANOVA on FOD +1 counts (ascending counting bias) revealed a main effect of Condition, *F*(2, 34) = 6.34, *p* = .005, η_p_^2^ = .27, but neither main effects of Group, *F*(2, 17)<1, nor a Condition×Group interaction, *F*(4, 34)<1. Exploratory analyses showed that the main effect of Condition was driven by LS participants, *F*(2, 14) = 5.52, *p* = .042, η_p_^2^ = .44; LDHS, *F*(2, 14)<1.5, and HDHS, *F*(2, 6)<2.1, participants did not differ across conditions. Relative to the baseline control condition, LS participants displayed reduced FOD +1 counts in the posthypnotic amnesia, *t*(7) = 2.89, *p* = .023, *d* = 0.94, and post-cancellation, *t*(7) = 4.43, *p* = .003, *d* = 0.47, conditions, which did not differ, *t*(7)<1.25. Relative to the simulated data, LS participants displayed lower FOD +1 counts in the posthypnotic amnesia condition, *t*(1006) = 2.51, *p* = .012, *d* = 0.89, but in neither of the control conditions, *t*s(1006)<1.4. LDHS participants' counts didn't differ from those of the simulated data in the control condition, *t*(1006)<1.5, but were significantly lower than the counts in the simulated data in the posthypnotic amnesia, *t*(1006) = 2.52, *p* = .012, *d* = 0.90, and the post-cancellation, *t*(1006) = 2.90, *p* = .004, *d* = 1.03, conditions. In contrast, HDHS participants' counts didn't differ from the simulated data in any of the conditions, *t*s(1002)<1.1. Cumulatively, these findings indicate that LS and LDHS, but not HDHS, participants exhibited an atypical reduction in descending counting in the posthypnotic amnesia condition; the latter group also displayed this effect in the post-cancellation condition.

## Discussion

Our results show that, in a subset of HS individuals, temporarily disrupting memory for previously generated numbers reduces repetition avoidance during RNG, thereby increasing the randomness of responses. In particular, we show that during posthypnotic amnesia HDHS, but neither LDHS nor LS, participants exhibited a selective increase in repetitions, resulting in equivalent performance to a purely random system. These results provide evidence that repetition avoidance during RNG stems from the retention of previous responses in working memory (see also [2], [3]). Our results also corroborate previous results indicating that baseline RNG performance is unrelated to hypnotic suggestibility [16]–[19].

The observed variability in responding among HS individuals is consistent with previous research. That the improvement in RNG during posthypnotic amnesia was only present in HDHS individuals fits with previous studies showing greater responsiveness to posthypnotic suggestions in this subgroup [9], [10]. Posthypnotic amnesia may augment normal forgetting through a top-down control process originating in the orbitofrontal cortex that disrupts the contents of working memory pertaining to previous responses [5]. Variegation among HS individuals thus may be attributable to superior cognitive control in HDHS individuals [20], which may facilitate the top-down mechanisms required to keep previous numerical responses from biasing responses [5], [21], [22]. LDHS participants, on the other hand, appear to have shifted from a balance at baseline between descending and ascending counting, neither of which differed from random responding, to an increase in the former, and concomitant decrease in the latter, in the posthypnotic amnesia condition. LS participants displayed a similar decrease in ascending counting in the posthypnotic amnesia condition. Insofar as these effects were specific to the posthypnotic amnesia condition, except for the continuation of the lower ascending counting to the post-cancellation condition in the LDHS participants, they appear to reflect these participants' attempts to respond to the posthypnotic suggestion and may point to similar mechanisms underlying responding in these two groups [23]. It is worth noting that both LDHS and HDHS participants reported selectively forgetting previous responses during the RNG task in the posthypnotic amnesia condition. Insofar as LDHS participants did not display a reduction in repetition avoidance, this may point to a dissociation between implicit and explicit processing in this group, as has often been observed during hypnotic responding in HS individuals more generally [24].

Notably, the posthypnotic amnesia suggestion did not reduce counting biases, probably because counting was not a prominent bias in the present sample at baseline. Alternatively, repetition avoidance may be a function of one's *conscious* memory of previous responses whereas counting biases reflect the inability to suppress over-learned number sequences and are less amenable to conscious control [25]. This interpretation is consistent with the observation that posthypnotic amnesia disrupts explicit memory while leaving implicit memory intact [6]. In the case of RNG, posthypnotic amnesia provides a unique instance in which forgetting confers a cognitive advantage and yields clear evidence that repetition avoidance depends upon the retention of previous responses in working memory [2], [3]. The approach utilized in this study could be exploited to examine further instances in which memory acts as an impedance to optimal cognitive functioning, such as in post-traumatic stress disorder.
